# Electrochemical Characterisation and Confirmation of Antioxidative Properties of Ivermectin in Biological Medium

**DOI:** 10.3390/molecules28052113

**Published:** 2023-02-24

**Authors:** Milan Selaković, Mara M. Aleksić, Jelena Kotur-Stevuljević, Jelena Rupar, Branka Ivković

**Affiliations:** 1 Faculty of Pharmacy, Department of Pharmaceutical Chemistry, University of Belgrade, 11351 Belgrade, Serbia; 2 Faculty of Pharmacy, Department of Physical Chemistry and Instrumental Methods, University of Belgrade, 11351 Belgrade, Serbia; 3 Faculty of Pharmacy, Department of Medical Biochemistry, University of Belgrade, 11351 Belgrade, Serbia

**Keywords:** ivermectin, voltammetry, glassy carbon electrode, oxidation and reduction mechanism, pro-oxidant, antioxidant and oxy-scores

## Abstract

Ivermectin (IVM) is a drug from the group of anthelmintics used in veterinary and human medicine. Recently, interest in IVM has increased as it has been used for the treatment of some malignant diseases, as well as viral infections caused by the Zika virus, HIV-1 and SARS-CoV-2. The electrochemical behaviour of IVM was investigated using cyclic (CV), differential pulse (DPV) and square wave voltammetry (SWV) at glassy carbon electrode (GCE). IVM showed independent oxidation and reduction processes. The effect of pH and scan rate indicated the irreversibility of all processes and confirmed the diffusion character of oxidation and reduction as an adsorption-controlled process. Mechanisms for IVM oxidation at the tetrahydrofuran ring and reduction of the 1,4-diene structure in the IVM molecule are proposed. The redox behaviour of IVM in a biological matrix (human serum pool) showed a pronounced antioxidant potential similar to that of Trolox during short incubation, whereas a prolonged stay among biomolecules and in the presence of an exogenous pro-oxidant (tert-butyl hydroperoxide, TBH) resulted in a loss of its antioxidant effect. The antioxidant potential of IVM was confirmed by voltametric methodology which is proposed for the first time.

## 1. Introduction

At the onset of the 2019 coronavirus pandemic (COVID-19), before any recognised effective treatment or currently approved vaccine therapy was available, an urgent need arose to re-evaluate existing medicines for the treatment of SARS-CoV-2 virus infection [1]. When IVM, a member of the avermectin family, demonstrated its antiviral potential against the virus [2], interest in IVM skyrocketed and there were numerous attempts to repurpose it for the treatment of COVID-19. IVM was first discovered in the 1970s by microbiologist Satoshi Omura and parasitologist William Campbell and is an anthelmintic drug [3]. IVM is a macrocyclic lactone derived from avermectins and is a mixture of two homologues: 22,23-dihydroavermectin B_1a_ (≥80%) and 22,23-dihydroavermectin B_1b_ (≤20%). It was first marketed for use in animals in 1981, but is also used to treat various diseases in humans, such as onchocerciasis [4]. Ivermectin is considered the drug of choice for various parasitic diseases [1]. IVM has been reported to be used in the treatment of some malignant diseases, as well as viral infections caused by the Zika and HIV-1 viruses and the SARS-CoV-2 virus [5].

Since the spike in interest, a number of electrochemical methods for the detection of IVM have been reported, such as the use of a boron-doped diamond (BDD) electrode cathodically pretreated with 0.5 mol L^−1^ H_2_SO_4_ for detection in pharmaceutical formulations and urine [6]; GCE modified with silver nanoparticles at modified boron and sulphur co-doped reduced graphene oxide nanohybrid (AgNPs B, S@rGO) for detection in injections, urine and tap water [7]; glutaraldehyde-modified GCE for detection in tap water and urine [8]; and β-cyclodextrin/graphene-based electrode for detection in tap water [9]. The BDD electrode provides a sensitive, simple, inexpensive and rapid amperometric flow injection method with results that show good agreement with comparable methods. However, the potentiostatic electrolyses resulted in fouling of the BDD electrode surface, which made it impossible to estimate the number of electrons involved in the process and to propose an oxidation mechanism of the IVM [6]. The modified electrode showed significantly increased sensitivity in IVM determination due to the synergistic effects of AgNPs and B, S@rGO. The proposed sensor exhibited good selectivity, reproducibility and long-term stability [7]. The glutardialdehyde-modified glassy carbon electrode showed high sensitivity, selectivity and stability [8], while the β-cyclodextrin/graphene-based electrode resulted in excellent analytical performance for the electrochemical detection of IVM [9].

None of the methods have characterised the electrochemical behaviour of IVM, none have been performed on a non-modified GCE and none of the methods for detecting IVM have been performed in human serum as biological material in which the distribution of IVM should be expected. Electrochemical characterisation of IVM was performed with the aim of better understanding the redox behaviour of the molecule, as well as predicting possible oxidation/reduction mechanisms and potential transformations during interaction with other electroactive biomolecules. Electrochemical methods are inexpensive, fast and easy to perform, making them ideal candidates for drug analysis.

Oxidative stress is the result of an imbalance in cellular antioxidant capacity and cellular levels of reactive oxygen species (ROS) and leads to damage in important cellular components such as DNA, proteins and lipids. The effects of oxidative stress are observed in a number of diseases, as well as in a variety of drug-induced toxicities [10].

Antioxidant activity is traditionally studied by well-known spectrophotometric methods, but they have some disadvantages. Very often, these methods require a lengthy and demanding pretreatment; especially for biological samples, the results may be affected by the turbidity of the sample. Therefore, the electrochemical approach could be very important as an independent and comparative method in the measurement of antioxidants and antioxidant activity due to its fast and simple methodology and the good stability of the electrochemical signal, which is not affected by turbid or opaque samples. Most studies using electrochemical approach in the measurement of antioxidant activity are used for the determination of polyphenolic compounds. Among them, the concept of electrochemical index (EI), defined as the total polyphenolic content obtained by nonselective oxidation of all polyphenols [11,12], is very promising because it uses the peak anodic potential and current to estimate the ease with which the compound is oxidized. In our study on IVM, a different approach was used—we employed DPV and used voltametric peak parameters to estimate the antioxidant properties of IVM in human serum samples. According to the order of occurrence of oxidation peaks, the following rule applies: the lower the oxidation potentials, the greater the ability of the compound to act as an electron donor, which is associated with higher antioxidant activity and antioxidant capacity.

The aim of this study is to investigate the redox activity of IVM in human serum pool of healthy individuals in order to compare it with a known antioxidant, Trolox (a water-soluble analogue of vitamin E), and to predict its pro-oxidant and antioxidant activity in vitro, as well as to propose which part of the chemical structure is responsible for the effect. A new electrochemical method to confirm the results of the oxidative stress testing is also proposed.

## 2. Results and Discussions

### 2.1. Electrochemical Characterisation

Preliminary tests were performed by CV in a 0.2 mmol L^−1^ IVM solution in 0.1 mol L^−1^ acetate buffer pH 4.6. Voltammograms were recorded in three consecutive scans, starting from 0.0 V to +1.6 V and back to −1.6 V; and from 0.0 V to −1.6 V and back to +1.6 V. Both voltammograms were recorded at a scan rate *ν* = 50 mV s^−1^.

**Figure 1:**
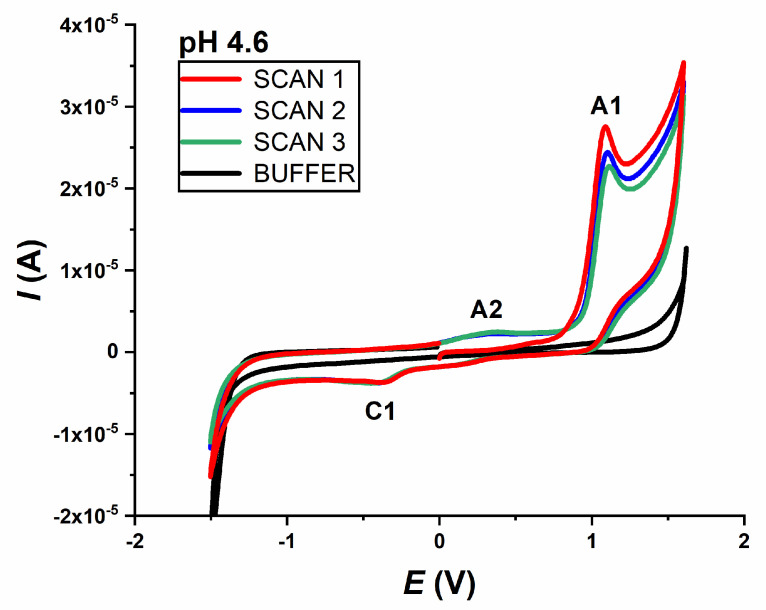
The CVs of 0.2 mmol L^−1^ IVM in acetate buffer pH 4.6, starting from 0.0 V, recorded in the positive potential direction; first (──), second (──), third (──) and buffer scan (──); *ν* = 50 mV s^−1^.

In the case where the potential was scanned towards the positive limit, two peaks appear in the first scan of the voltammogram: one main anodic peak (A1) at a potential of about +1.0 V, which decreases with the subsequent scans, and one cathodic peak (C1) at a potential of about −0.5 V, which does not change. In the second scan, another anodic peak (A2) appears at a potential of about +0.3 V, indicating that it was detected as an oxidation peak of the previously reduced form of the drug (Figure 1).

When the scan direction was changed and scanned from 0.0 V to −1.6 V and back to +1.6 V (not shown), IVM again revealed one cathodic peak (C1 at about −0.5 V), but in this case two anodic peaks (A2 at +0.3 V and A1 at +1.0 V), both in the first scan. The appearance of the A2 peak (when scanning in this direction) in the first scan is probably due to the strong negative potential applied previously, which caused the formation of the reduced form of the drug that can be further be oxidised. It should be noted that the intensity of the oxidation peak A2 is very weak compared to the main oxidation product (A1 peak).

All recorded CVs of IVM at different pH values (pH range 2–10) showed the same behaviour. The irreversibility of the oxidation and reduction processes was also confirmed, as both the anodically and cathodically recorded peaks did not show a corresponding reversal response.

### 2.2. Oxidation

**Figure 2:**
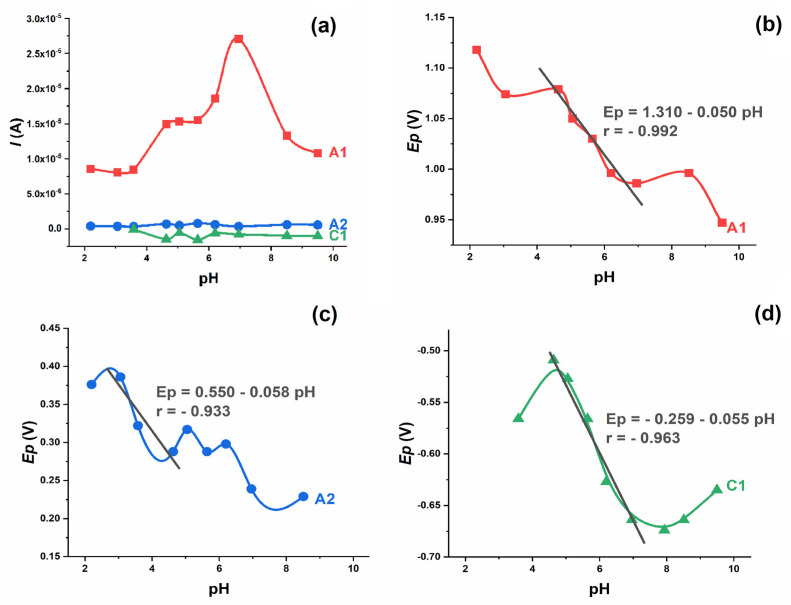
The influence of pH on the 0.2 mmol L^−1^ IVM CV peak current and potential. Plot of *I*_p_ vs. pH ■—peak A1, ●—peak A2, ▲—peak C1 (**a**). Plot of *E*_p_ vs. pH for peak A1 (**b**), peak A2 (**c**) and peak C1 (**d**).

The oxidation of 0.2 mmol L^−1^ IVM and the corresponding anodic peak (A1) were observed in the supporting electrolytes at different pH values from 2.0 to 10.0 (Figure 2a). The oxidation peak exists at all pH values studied and the peak potential shifts to less positive values with the increasing pH value. Both the potential and the intensity of the A1 peak are pH dependent. The intensity of the peak is highest around pH 7.0, i.e., in neutral solutions. The dependence of *E*_p_ vs. pH shows the characteristic “S” shaped curve, with linear dependence in the pH range 4–8, which follows the equation (Figure 2b):

*E*_p,A1_ = 1.310 V − 0.050 pH. (1)

The slope of 50 mV/pH indicates that the proton–electron ratio is 1:1, which means that the same number of protons and electrons are involved in the electrode process [13].

From the difference between the peak potential and the peak potential at half height for irreversible electrode processes—defined by the equation

(2) $$\left|E_{p,A1}-E_{p1/2,\;A1}\;\right|=\frac{47.7}{\alpha_{n}n},$$

where the charge transfer coefficient $\alpha_{n}$ can be approximately 0.5—the number of electrons transferred in the rate-determining step (n) can be calculated [14]. In the case of the A1 peak, the above-mentioned potential difference was between 80 and 100 mV (depending on the pH), which indicates that one electron is involved in the reported oxidation process.

To investigate the influence of scan rate, CVs were recorded with a concentration of 0.2 mmol L^−1^ IVM at pH 4.6 and pH 6.9 at different scan rates ranging from 10 to 100 mV s^−1^. A linear dependence of *I*_p,A1_ vs. the square root of the scan rate (*ν*^1/2^) was observed at both pH values. The regression equations

*I*_p_ = 7.361 × 10^−7^ + 1.112 × 10^−4^ *ν*^1/2^, (3)

with a correlation coefficient of r = 0.998 (pH 4.6), and

**Figure 3:**
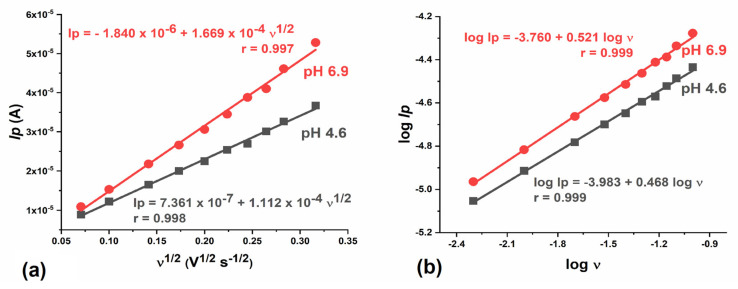
The effect of scan rate on the peak current of oxidation peak A1 peak. (**a**) Plot of *I*_p,A1_ vs. *ν*^1/2^; (**b**) Plot of log *I*_p_ vs.log ν. pH = 4.6 (──), pH = 6.9 (──).

*I*_p_ = −1.840 × 10^−6^ + 1.669 × 10^−4^ *ν*^1/2^, (4)

with a correlation coefficient of r = 0.997 (pH 6.9)—were determined (Figure 3a). The obtained value of the correlation coefficient, which is close to unity, indicates that IVM oxidation is a diffusion-controlled reaction in mild acidic and neutral solutions. A value of the intercept that slightly deviates from 0 (at both pH values) suggests that the electrode process is preceded by a chemical reaction, i.e., proton transfer.

A linear dependence of log *I*_p,A1_ vs. log *ν* was observed at both pH values (Figure 3b). The corresponding slopes were always close to the theoretical value of 0.5, confirming that the oxidation process of IVM in mild acidic and neutral solutions at the GCE is a diffusion-controlled process.

**Figure 4:**
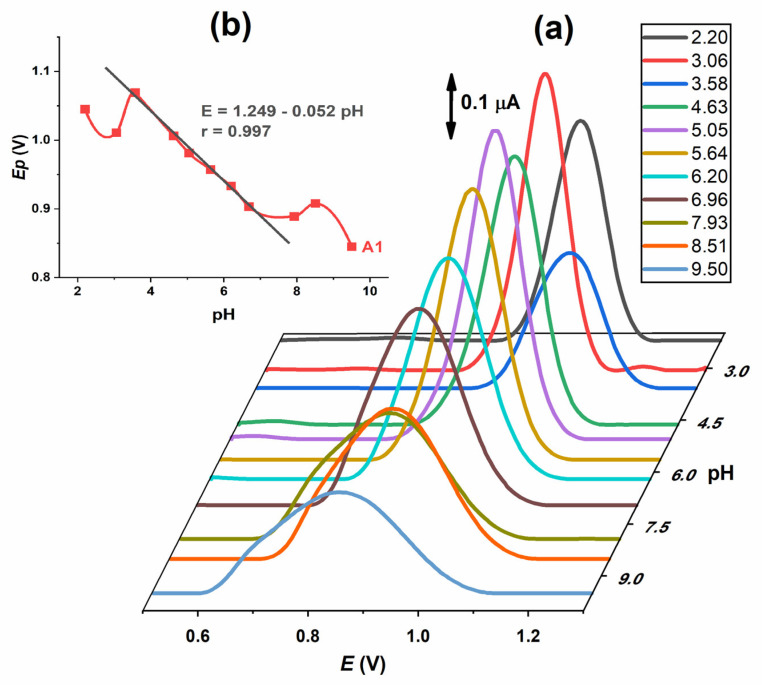
(**a**) 3D plot of DP voltammograms of 0.2 mmol L^−1^ IVM recorded at different pH values. (**b**) Plot of *E*_p_ vs. pH for oxidation A1 peak.

DP voltammograms of 0.2 mmol L^−1^ IVM solution were recorded in the same supporting electrolytes as for CV at various pH values from 2.0 to 10.0. One well-developed oxidation peak (A1) was observed (Figure 4a). Both the potential and the intensity of the A1 peak are pH-dependent. The intensity of the peak is highest at pH 5.0–6.0. The potential decreases with the increase in pH, with a linear part of the curve in the range 3.5–7.5 with the slope of Δ*E*_p_*/*ΔpH = 52 mV (Figure 4b). When the half peak width (W_1/2_) was analysed, it was found that its value is approximately 90–120 mV at pH ≤ 7 and increased with pH. According to the results of the pH influence on IVM DP voltammograms, the conclusions from CV are confirmed as follows: the oxidation process, represented by the A1 peak, is pH-dependent and proceeds with the exchange of one electron and one proton.

SW voltammograms of 0.2 mmol L^−1^ IVM solution were also recorded in supporting electrolytes at various pH values from 2.0 to 10.0. The same well-developed oxidation peak (A1) (not shown) as in DP voltammograms was observed. As stated, both the potential and the intensity of the A1 peak are pH-dependent. The intensity of the peak is highest at pH 5.0. The peak potential decreases as the pH increases. Since this technique registers both oxidation and reduction currents in the same experiment, the irreversible nature of oxidation was confirmed by analysing the forward and reverse current components of the A1 peak. 

The second oxidation peak (A2) was only visible in the second and third scans (Figure 1), indicating that it originates from the oxidation of the previously reduced form of the drug. The potential of the A2 peak is pH-dependent and shifts to more negative values with the increasing pH, while the intensity is not pH-dependent. As stated, an “S”-shaped curve was obtained with a linear segment in the narrow pH range (3–5), where the dependence of *E*_p_ vs. pH followed the equation (Figure 2c):

*E*_p,A2_ = 0.550 V − 0.058 pH. (5)

The slope of 58 mV indicates that an equal number of electrons and protons are involved in the electrode process. The A2 peak was not visible in DP and SW voltammograms.

### 2.3. Reduction

The reductions of IVM and the corresponding cathodic peak (C1) were observed in supporting electrolytes at different pH values (Figure 2d). In an acidic environment, it is absent; at pH 3.5, it is weak and hardly visible; and with a further increase in pH, the C1 peak increases with uniform intensity. At pH 4.5 < pH < 7.5, a linear dependence of the peak potential on pH was determined according to the following equation:

*E*_p,C1_ = −0.259 V − 0.055 pH. (6)

The slope of 55 mV indicates that the number of protons involved in the electrode process is equal to the number of electrons. In more alkaline solutions (pH > 7.5) the C1 peak potential flattens out and the slope *E*_p_ vs. pH decreases significantly, indicating that the reduction process does not involve proton transfer under these conditions.

Since the differences between peak potential and peak potential at half-height ($\left|E_{p,C1}-E_{p1/2,\;C1}\;\right|$) were −0.095 V (pH 4.6) and −0.097 (pH 6.9), it is indicated that one electron is exchanged in the reduction process represented by the reduction peak C1 [14].

To investigate the influence of scan rate, CVs were recorded with a concentration of 0.2 mmol L^−1^ IVM at pH 4.6 and pH 6.9 at different scan rates of 10–100 mV s^−1^. A linear dependence of *I*_p,C1_ vs. the scan rate (*ν*) was observed at both pH values. The regression equations (Table 1) provide a high correlation coefficient of r > 0.99, indicating that IVM reduction is an adsorption-controlled reaction in acidic and neutral solutions. As previously mentioned, a value of the intercept that slightly deviates from 0 suggests that the electrode process at both pH values is preceded by a proton transfer. Moreover, the linear dependence of log *I*_p_ vs. log *ν* was observed at both pH values (Table 1). The corresponding high slopes close to unity confirm that the reduction process of IVM at GCE is controlled by adsorption.

### 2.4. Reaction Mechanism

**Figure 5:**
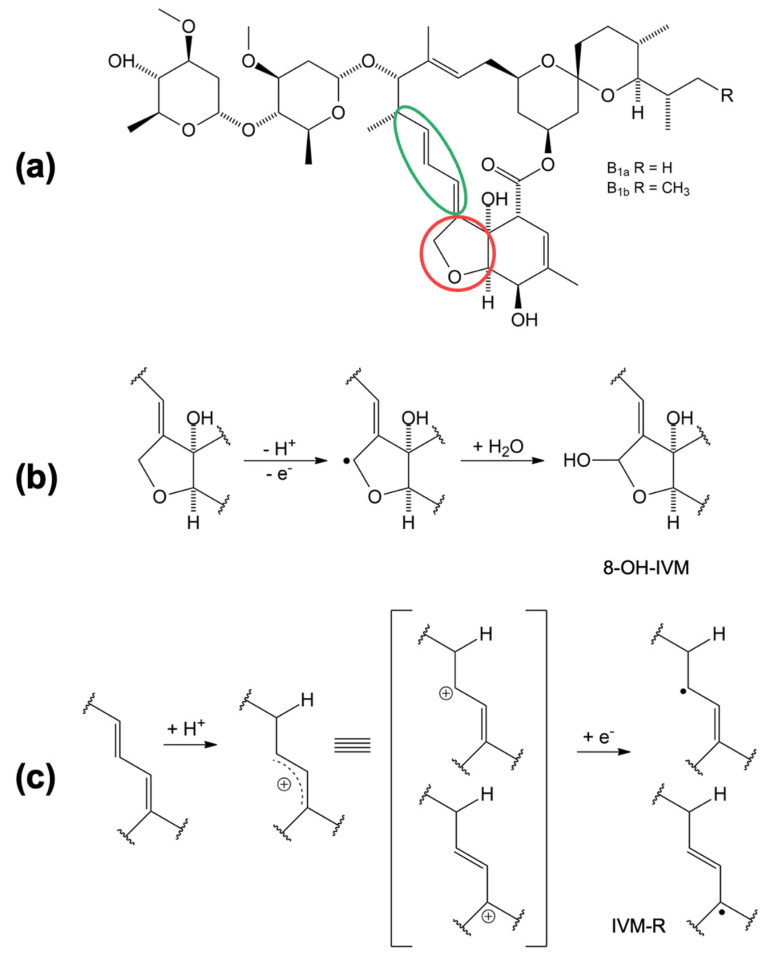
Proposed oxidation and reduction reaction mechanisms of IVM; (**a**) IVM structure with oxidation site marked in red and reduction site marked in green; (**b**) oxidation mechanism; (**c**) reduction mechanism.

Based on the electrochemical data obtained and presented, a mechanism of oxidation and reduction is proposed in Figure 5. The oxidation and reduction sites on the IVM structure (Figure 5a) are marked in red and green, respectively. The oxidation takes place at position 8A in the tetrahydrofuran (THF) ring [15]. In the first step, IVM forms an intermediate radical (8-ivermectin radical) in contact with the electrode, which further reacts with the OH radical from the water to form 8-hydroxy ivermectin (8-OH IVM) as the main product (Figure 5b). Considering the experimentally obtained results, we assume that the reduction process of IVM takes place at the 1,4-diene structure in slightly acidic and neutral medium. In the first step, a resonantly stabilised carbocation is formed by protonation, which receives an electron and forms the product IVM-R (Figure 5c).

### 2.5. Antioxidant Properties

IVM stock samples were prepared by dissolving in dimethyl sulfoxide (DMSO) or in a mixture of polyethylene glycol and glycerol (PEG/Gly) in a 6:1 ratio. Two sets of serum pool samples were prepared. The first set was incubated for 2 h at 37 °C, and the second for 24 h to mimic the internal conditions of the organism. After incubation, redox status parameters were measured in the prepared serum pool samples: serum pro-oxidant-antioxidant balance (PAB) and serum total oxidant status (TOS) (as representatives of pro-oxidants), and total antioxidant status (TAS) and levels of total sulfhydryl groups (SHG) (as representatives of antioxidants). Z-scores were calculated from the measured parameters to estimate the difference between the redox status of the pool of natural serum samples and the addition of IVM stock samples. The Z-score values enabled the pro-oxidant score (PS, as the average Z-score value of TOS and PAB Z-scores), the antioxidant score (AOS, as the average value of TAS and SHG Z-score values). The oxy-score (OS) was calculated as the difference between the values of PS and AOS. The results are shown in Table 2 and Table 3.

**Figure 6:**
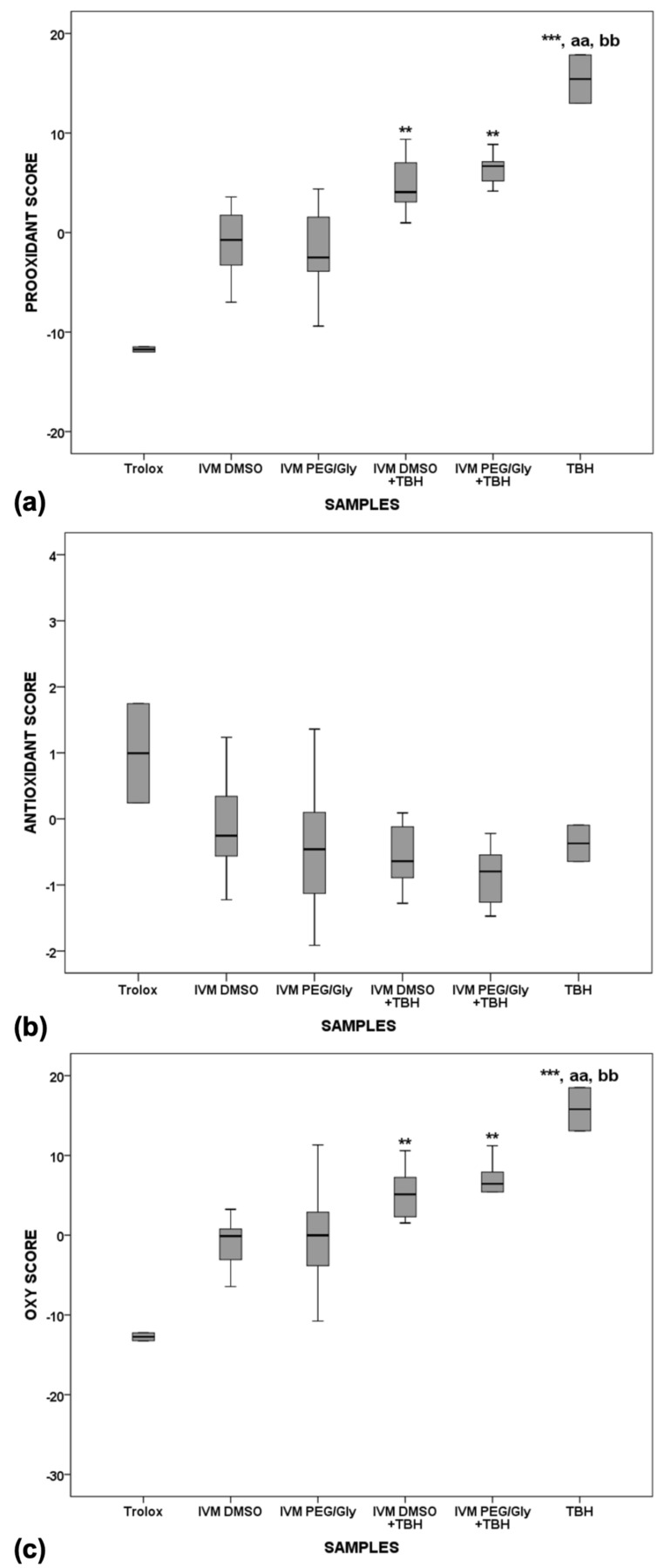
Pro-oxidant (**a**), antioxidant (**b**) and oxy-scores (**c**) after 2 h of incubation. ** *p* < 0.01 vs. Trolox; *** *p* < 0.001 vs. Trolox; aa *p* < 0.01 vs. IVM DMSO; bb *p* < 0.01 vs. IVM PEG/Gly.

The presence of dose-dependent activity was tested with 5 different dilutions in 2 different solvents. Since no significant change was found in the measured parameters, the results presented are the average of all 5 different concentrations. Trolox, which was used as a strong representative of the antioxidants in serum, showed the lowest pro-oxidant and highest antioxidant activity (Figure 6a,b), whereas TBH, which was used as a strong representative of the pro-oxidants, shows the highest pro-oxidant activity in serum (significantly higher than the pro-oxidant activities of Trolox, *p* < 0.01). Both IVM solutions showed similar antioxidant properties to Trolox after 2 h of incubation in serum. When TBH is added to serum with the IVM solutions, the oxy-score is significantly higher after 2 h of incubation compared with Trolox (Figure 6c, *p* < 0.001), indicating that the antioxidant effect of IVM is present but not sufficient to counteract the potent pro-oxidant effect of TBH.

**Figure 7:**
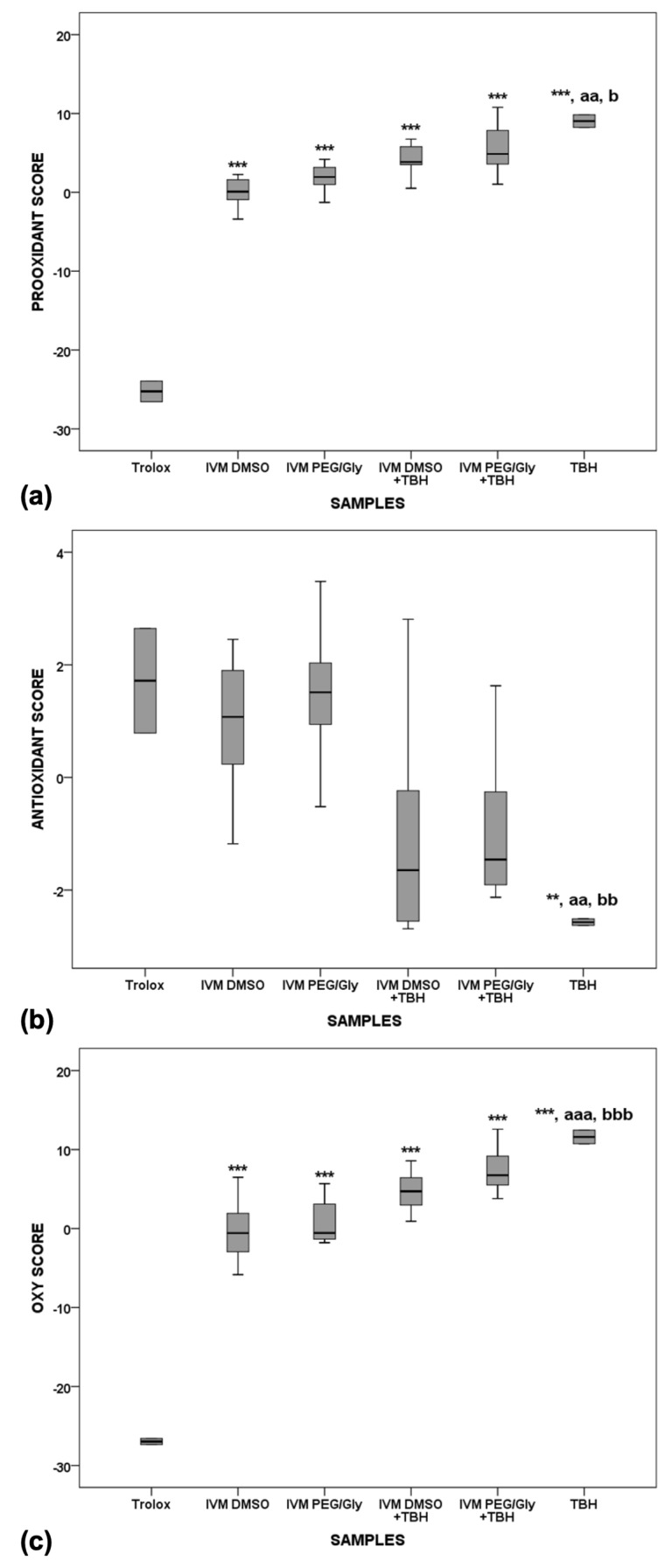
Pro-oxidant (**a**), antioxidant (**b**) and oxy-scores (**c**) after 24 h of incubation. ** *p* < 0.01 vs. Trolox; *** *p* < 0.001 vs. Trolox; aa *p* < 0.01 vs. IVM DMSO; aaa *p* < 0.001 vs. IVM DMSO; b *p* < 0.05 vs. IVM PEG/Gly; bb *p* < 0.01 vs. IVM PEG/Gly; bbb *p* < 0.001 vs. IVM PEG/Gly.

After the 24-h incubation (Figure 7), Trolox alone showed the lowest oxidative effect in serum compared with IVM solutions alone (*p* < 0.001), IVM solutions with TBH (*p* < 0.001), and TBH alone in serum (*p* < 0.001). TBH alone in serum still shows the highest pro-oxidant potential and a statistically significant difference is observed compared to Trolox alone, as well as to both IVM solutions alone in serum. While there is a significant difference between Trolox and IVM solutions when comparing the oxidative and prooxidative values, no difference is observed when comparing the antioxidant value, which means that the behaviour of IVM, which is more similar to Trolox, does not change over time.

When comparing the pro-oxidant, antioxidant and oxy-scores of samples incubated for 2 h versus 24 h, a statistically significant difference (*p <* 0.01) was observed between the pro-oxidant and antioxidant values (both values were higher after the 24-h incubation) of the IVM solution in PEG/Gly, but no difference was observed in the oxy-scores of the solutions, implying that the differences cancel each other out. Since no difference was observed in the IVM solutions in DMSO, this could be the result of a synergistic effect between the antioxidant effect of DMSO and IVM, which means that IVM solutions in PEG/Gly need more time to develop their effect. However, the use of DMSO up to 0.5 mL has not shown any effect on antioxidant assays, although it could be an antioxidant under certain conditions and/or concentrations [16].

**Figure 8:**
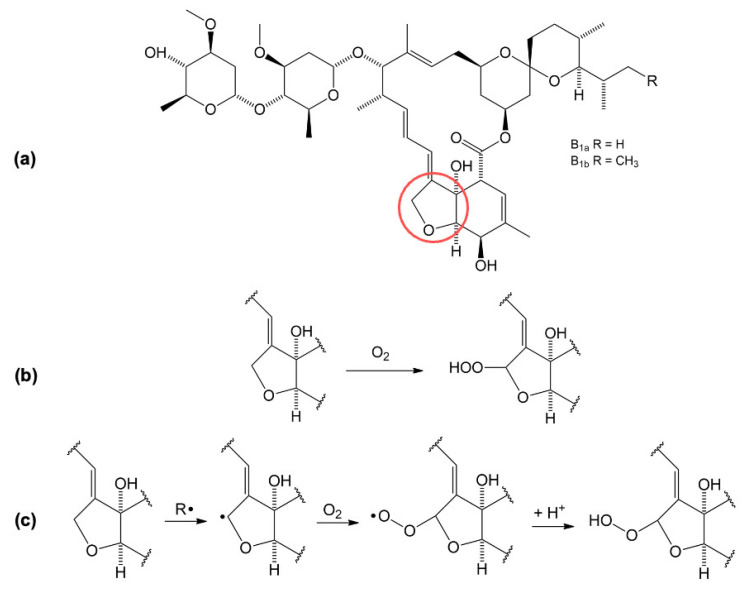
Oxidation reaction and reaction mechanism of IVM in serum; (**a**) IVM structure with oxidation site marked in red; (**b**) oxidation reaction; (**c**) oxidation reaction mechanism.

Since it has been concluded that IVM has antioxidant activity, a proposed reaction for the oxidation of IVM is shown in Figure 8. Oxidation occurs in a reaction with oxygen (O_2_) dissolved in the serum at the THF ring in position 8A (marked in red) and an unstable peroxide is formed [15].

### 2.6. Electrochemical Analysis of the Antioxidative Properties

**Figure 9:**
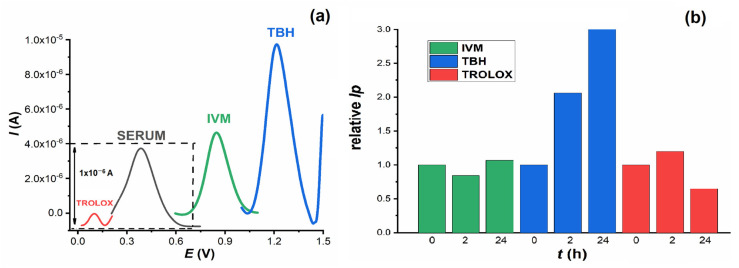
(**a**) DP baseline corrected voltammograms of blank human serum (──), Trolox in human serum (──); IVM in human serum (──) and TBH in human serum (──). (**b**) Relative intensity of IVM, TBH and Trolox oxidation peaks immediately after addition to serum (*t* = 0), after 2 h and 24 h incubation at 37 °C.

To confirm the results of the spectrophotometric measurements, voltametric DP measurements were carried out. In a small voltametric cell (volume 1.5 mL), DP voltammograms were recorded from blank serum pool samples and from serum with the addition of IVM, Trolox and TBH. Measurements were taken immediately after addition and after 2 h and 24 h incubation at 37 °C. All investigated substances can be oxidised at the GCE and give oxidation peaks at different potentials: Trolox at *E*_p_ = 0.08 V, serum at *E*_p_ = 0.40 V, IVM at *E*_p_ = 0.85 V and TBH at *E*_p_ = 1.255 V (Figure 9a). Considering the increasing values of the oxidation potential of these compounds, it can be assumed that Trolox is the easiest and TBH the most difficult to oxidise, which is consistent with the fact that Trolox has a higher antioxidant effect than TBH and is a well-known antioxidant.

**Figure 10:**
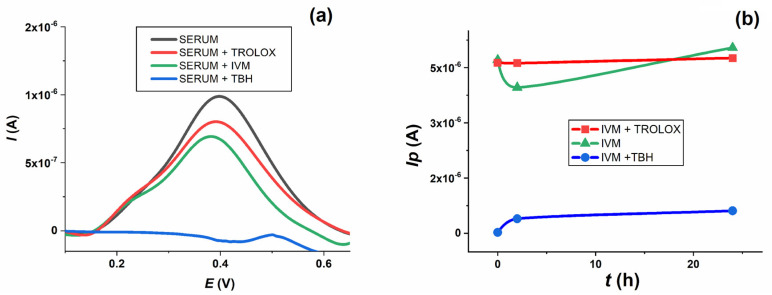
(**a**) Baseline corrected DPV of blank human serum oxidation, immediately (──) and after addition of Trolox (──), IVM (──) and TBH (──) after 24 h of incubation. (**b**) Change in oxidation peak current of IVM during 24 h of incubation in human serum (──), and human serum with added Trolox (──) and TBH (──).

Since the oxidation of IVM has already been confirmed, the aim of further experiments was to study its behaviour after incubation in serum samples and compare it with Trolox and TBH. As can be seen in Figure 9b, the intensity of the TBH oxidation peak increased sharply with increasing incubation time. In contrast, IVM and Trolox showed no significant change. It is interesting to follow the change in serum oxidation peak after addition and incubation of all compounds (Figure 10a). When TBH was added to the serum sample, its oxidation peak at about 0.4 V disappeared almost immediately and was no longer seen in the voltammogram when incubated for up to 24 h. In the presence of Trolox and IVM, the height of the serum oxidation peak decreased to about 80% and 70 % of its original height, respectively.

When TBH was added to the serum along with the IVM solutions, the oxidation peak of IVM (green line) decreased dramatically (blue line), whereas the level of the oxidation peak of IVM changed only insignificantly when Trolox was added (red line) throughout the 24 h incubation period (Figure 10b). The effect of TBH on IVM oxidation could be explained by the fact that TBH, as a strong oxidising agent, completely oxidises the IVM present in the serum sample, leaving nothing behind that could be subsequently oxidised at the electrode. Trolox, on the other hand, as a reducing agent, has no effect on the IVM oxidation process. The summary of the results presented suggests that IVM has similar antioxidant properties to Trolox after incubation in human serum samples, which is consistent with the spectrophotometric measurements and the corresponding oxy-score of IVM, based on the calculated pro-oxidant and antioxidant scores.

## 3. Materials and Methods

### 3.1. Chemicals

IVM was obtained from Horster Biotek Pvt. Ltd. India (serial number: 21,005 HBL, LOT: IVR/20-21/011, expiration date: 01.2025.). A stock solution of IVM (2 mmol L^−1^) for electrochemical characterisation was prepared in methanol. Stock solutions of IVM (0.25 mmol L^−1^) for the biochemical assays were prepared by dissolving IVM in DMSO or PEG/Gly mixture in a 6:1 ratio. 

Solutions of different concentrations for electrochemical characterisation were obtained by diluting the stock solution with different supporting electrolytes prepared with chemicals of analytical-grade quality. The following supporting electrolytes were used: citric acid/sodium hydroxide buffer (pH 2.20, 3.06), acetate buffer (pH 3.58, 4.63, 5.05, 5.64), phosphate buffer (pH 6.20, 6.96, 7.93) and ammonia/ammonium chloride buffer (pH 8.51, 9.50) [17]. The ionic strength of all solutions was adjusted to 0.1 mol L^−1^. All experiments were performed at room temperature (25 ± 1 °C). 

The serum pool was prepared from serum samples of healthy individuals and kindly donated by the Military Medical Academy in Belgrade after daily work in the biochemical laboratory, which would otherwise be destroyed as biological waste. Aliquots of the serum pool were frozen at −80 °C and analysed within 5 days of collection.

Biochemical analysis: All samples were incubated in duplicate for 2 and 24 h at 37 °C. Serum pool samples (450 μL) were mixed in 1:10 ratio with different solutions (50 μL) as follows: Trolox (2 mmol L^−1^), TBH (0.25 mmol L^−1^) and dilutions of IVM stock solution (0.25–0.015625 mmol L^−1^). The IVM samples were mixed in equal parts with TBH to test its reactivity in the presence of exogenous pro-oxidants (exactly 25 μL were added to the serum pool samples).

Electrochemical assays: Serum pool samples (1.35 mL) were mixed in a 1:10 ratio with different solutions (0.15 mL) as follows: Trolox (2 mmol L^−1^), TBH (0.25 mmol L^−1^), IVM (2 mmol L^−1^ stock solution in methanol), IVM + Trolox (75 μL each) and IVM + TBH (75 μL each). Voltammograms were recorded after addition (*t* = 0 h), after 2 h and after 24 h incubation at 37 °C.

### 3.2. Apparatus

A μAUTOLAB analyser (EcoChemie, Utrecht, The Netherlands) and GPES 4.9 software were used for voltametric measurements. A three-electrode system was used in all experiments: GCE (CH Instruments, Inc., Austin, TX, USA, d = 3 mm) as the working electrode, an Ag/AgCl as the reference electrode (3 M KCl) and a Pt wire as the auxiliary electrode. pH measurements were performed using a PHM 220 pH meter with a combined electrode Radiometer GK240B. Sonication was performed in an ‘Iskra’ UZ 4R ultrasonic bath (Sentjernej, Slovenia) and a SCALTEC SBC 31 balance was used for weight measurements. Spectrophotometric measurements were performed using the SPECTROstar Nano Microplate Reader (BMG Labtech, Ortenberg, Germany).

### 3.3. Procedures

Electrochemical characterisation was performed by CV, DPV and SWV. The effect of pH was studied in the pH range of 2–10. The GCE was manually polished with an aqueous slurry of Al_2_O_3_ powder (particle size 0.05 μm) on a smooth polishing pad for 2 min before each experiment. The electrode was rinsed with deionised water and then sonicated in deionised water for 2 min and in absolute ethanol for 2 min and then rinsed with deionised water. The test solutions were deoxygenated by bubbling with high-purity nitrogen before and in-between runs to remove oxygen interference. 

CV was first performed in the direction of the positive potential—starting at 0 V, increasing to +1.6 V, and decreasing again to −1.6 V—and in the opposite direction towards negative potential—starting at 0 V, decreasing to −1.6 V and increasing to +1.6 V. The scan rate was adjustable and ranged from 0.01 V s^−1^ to 0.1 V s^−1^, with a step potential of 0.005 V. DPV was used under the following conditions: step potential 0.005 V, modulation amplitude 0.05 V, modulation time 0.05 s and interval time 1 s. The conditions for SWV were frequency 25 Hz, step potential 0.001 V, effective scan rate of 0.025 V s^−1^ and pulse amplitude of 0.005 V. The DP and SW voltammograms shown were baseline-corrected by applying the moving average with a step window of 0.005 V. The corrected voltammograms were used only for better visualisation and graphical representation of peaks. All peak current values used and shown in all graphs were obtained from the original, untreated voltammograms.

PAB was measured according to a previously published method [18,19]. 3,3′-5,5′-Tetramethylbenzidine (TMB) reacts with both hydrogen peroxide and the antioxidants present in the sample. The reaction of chromogen and H_2_O_2_ is enzymatically catalysed by peroxidase, whereas the reaction of chromogen and antioxidants is not. Different proportions (0–100%) of 1 mmol L^−1^ H_2_O_2_ were mixed with 6 mmol L^−1^ uric acid to obtain the standard solutions. The results are expressed in arbitrary units corresponding to the percentage of H_2_O_2_ in the standard solution. TOS was determined according to the Erel’s method [20], which was optimised for our laboratory [18]. The ferrous ion-o-dianisidine complex is oxidised to ferric ion in presence of oxidants. The concentration of oxidant molecules is proportional to the colour intensity of the sample. TOS values are given as μmol H_2_O_2_ equivalent L^−1^, since the method was calibrated with 10–200 μmol hydrogen peroxide aqueous solution. SHG were measured using the optimised Ellman’s method [18,21]. The reaction between the reagent dinitrodithiobenzoic acid (DTNB, conc. 10 mmol L^−1^) and aliphatic thiol compounds in a basic environment (pH 9.0) provides 1 mol of p-nitrophenol anion per 1 mol of thiol. Method calibration was performed with reduced glutathione (conc. 0.1–1.0 mmol L^−1^). TAS was determined by Erel’s method [22] modified for our laboratory [18]. Reduced 2,2-azino-bis(3-ethyl-benz-thiazoline-6-sulfonic acid) (ABTS) oxidises in the presence of hydrogen peroxide in acidic medium. Antioxidants present in the sample lead to ABTS discoloration proportional to their concentrations. The reaction was calibrated with 6-hydroxy-2,5,7,8-tetramethylchroman-2-carboxylic acid (Trolox) and the results are expressed as μmol Trolox equivalent L^−1^. The oxidative score was calculated as the difference between the pro-oxidant score (TOS and PAB, i.e., average of Z-scores of all measured pro-oxidants and their products) and the antioxidant score (TAS and SHG, i.e., average of Z-scores of all measured antioxidants and their products). The difference between the original value and the control value divided by the standard deviation of the control values (or population means and standard deviations) gives the Z-score. The OS value is directly proportional to the pro-oxidant effect [23,24].

Statistical analysis was performed with SPSS 18.0 (SPSS inc. Chicago, IL, USA). Data are presented as medians ± interquartile range. The Friedmann’s test (nonparametric repeated-measures ANOVA) followed by Wilcoxon’s paired-samples (post hoc test for related samples) and Mann–Whitney U test were used to test the difference between quantitative variables. A significant difference was considered at *p <* 0.05 for each statistical test performed.

Electrochemical confirmation of the spectrophotometric analysis was performed by DPV in a small electrochemical cell (1.5 mL) using the same three-electrode system. The GCE was prepared in the same manner as described previously. Samples were tested without deoxygenation before and between runs. Voltammograms were recorded after 0 h, 2 h and 24 h incubation at 37 °C. DPV was used under the following conditions: step potential 0.005 V, modulation amplitude 0.05 V, modulation time 0.05 s and interval time 1 s. All values determined and presented were obtained from the original voltammograms. Baseline-corrected voltammograms (same procedure as described above) were used for display and visualisation only.

## 4. Conclusions

Electrochemical characterisation of IVM, a ratified anthelmintic but also a potential antitumor and antiviral drug, was performed using CV, DPV and SWV. IVM can be oxidised and reduced at GCE separately. Both the potential and intensity of the oxidation (A1 and A2) and reduction (C1) peaks are pH dependent. The influence of pH was studied in different buffer solutions in the pH range 2–10. The oxidation process, represented by the A1 peak, takes place as an irreversible, diffusion-controlled process involving the exchange of one electron and one proton in slightly acidic and neutral solutions. The oxidation of IVM occurs at position 8A in the tetrahydrofuran ring, forming an intermediate radical (8-ivermectin radical) that further transitions to 8-OH IVM as the major product. The reduction of IVM is an irreversible, adsorption-controlled process that occurs at the 1,4-diene structure. Upon protonation, a resonance-stabilised carbocation is formed, which, after the consumption of an electron, forms the reduced IVM-R product.

The antioxidant activity of IVM was studied in vitro in a human serum pool and compared with a known antioxidant (Trolox) and pro-oxidant (TBH). PAB, TOS, TAS and SHG were determined spectrophotometrically and used to calculate pro-oxidant, antioxidant and oxy-scores of IVM after 2 h and 24 h of incubation. The redox behaviour of IVM showed pronounced antioxidant potential, similar to that of Trolox at short-term incubation, whereas a prolonged stay among biomolecules, e.g., in the presence of an exogenous pro-oxidant (TBH), resulted in a loss of antioxidant activity. DPV measurements performed in an oxygenated human serum sample enriched with IVM, Trolox and TBH after incubation for up to 24 h at 37 °C revealed that IVM exhibited redox properties similar to Trolox and confirmed the results obtained by spectrophotometric measurements.

The electrochemical characterisation of IVM was performed with the aim of better understanding the redox behaviour of the molecule itself and predicting possible transformations during interactions with biomolecules in the biological matrix. The presented results are not only of fundamental importance, but also promote the use of electrochemical methods in addition to the known and accepted methods for the analysis of the antioxidant properties of drugs.

## Figures and Tables

**Table 1:** Regression equations and correlation coefficients of the *I*_p_ = *f* (*ν*) and log *I*_p_ = *f* (log *ν*) linear dependences, in acidic and neutral medium.

pH	*I*_p_/A = *f* (*ν*/V s^−1^)	r	log *I*_p_ = *f* (log *ν*)	r
4.6	−6.934 × 10^−8^ − 2.269 × 10^−5^ ν	−0.995	−4.667 + 0.958 log *ν*	0.997
6.9	4.551 × 10^−8^ − 1.502 × 10^−5^ ν	−0.996	−4.852 + 1.008 log *ν*	0.993

**Table 2:** POS, AOS and OS of IVM after 2 h.

Parameter	IVM DMSO	IVM PEG/Gly	IVM DMSO+ TBH	IVM PEG/Gly+ TBH	*p* *	Trolox	TBH
POS	−0.73 (−3.27–1.74)	−2.51 (−3.89–1.55)	4.07 (3.07–7.01)	6.02 (4.16–7.11)	0.001	a, b, c	a, b, c, d
AOS	−0.25 (−0.56–0.34)	−0.46 (−1.13–0.10)	−0.64 (−0.89–(−)0.12)	−0.80 (−1.26–(−)0.55)	0.197	d	ns
OS	−0.11 (−3.07–0.77)	−0.02 (−3.82–2.87)	5.12 (2.32–7.25)	6.44 (5.42–7.92)	0.008	a, b, c	a, b, c, d

* *p* from Friedman’s test for samples with 2 h incubation; ns = non-significant; a *p* < 0.05 vs. IVM DMSO; b *p* < 0.05 vs. IVM PEG/Gly; c *p* < 0.05 vs. IVM DMSO + TBH; d *p* < 0.05 vs. IVM PEG/Gly + TBH.

**Table 3:** POS, AOS and OS of IVM after 24 h.

Parameter	IVM DMSO	IVM PEG/Gly	IVM DMSO+ TBH	IVM PEG/Gly+ TBH	p *	Trolox	TBH
POS	0.09 ^cc^ (−0.94–1.60)	1.94 ^d^ (0.98–3.17)	3.48 ^aa^ (3.50–5.78)	4.87 ^b^ (3.59–7.86)	0.009	a, b, c, d	a, b, c
AOS	1.07 (0.24–1.90)	1.51 (0.94–2.03)	−1.65 (−2.55–(−)0.24)	−1.46 (−1.91–(−)0.26)	0.012	ns	a, b, d
OS	−0.57 ^cc^ (−2.94–1.93)	−0.56 ^dd^ (−1.33–3.10)	4.72 ^aa^ (2.97–6.44)	6.76 ^bb^ (5.52–9.18)	0.001	a, b, c, d	a, b, c

* *p* from Friedman’s test for samples with 2 h incubation; ns = non-significant; a, aa *p* < 0.05, 0.01, respectively, vs. IVM DMSO; b, bb *p* < 0.05, 0.01, respectively, vs. IVM PEG/Gly; c, cc *p* < 0.05, 0.01, respectively, vs. IVM DMSO + TBH; d, dd *p* < 0.05, 0.01, respectively, vs. IVM PEG/Gly + TBH.

## Data Availability

Not applicable.

## Sample Availability

Samples of the compounds are available from the authors.
